# Predicting atrial fibrillation and flutter using BEHRT and identifying multimorbidity patterns using BERTopic

**DOI:** 10.3389/fdgth.2026.1722338

**Published:** 2026-02-05

**Authors:** Sookyung Bae, Yeonjae Kim, Samina Park, Hwiyoung Kim, Bomi Park

**Affiliations:** 1 Department of Integrated Medicine, Yonsei University College of Medicine, Seoul, Republic of Korea; 2 Department of Preventive Medicine, Chung-Ang University College of Medicine, Seoul, Republic of Korea; 3 Department of Thoracic and Cardiovascular Surgery, Seoul National University College of Medicine, Seoul National University Hospital, Seoul, Republic of Korea; 4 Department of Neurosurgery, Yonsei University College of Medicine, Seoul, Republic of Korea

**Keywords:** atrial fibrillation and flutter, BEHRT, BERTopic, deep learning, multimorbidity

## Abstract

**Introduction:**

Atrial fibrillation and flutter are heart rhythm disorders frequently associated with multiple other chronic conditions, complicating their management and requiring optimized care. Analyzing pre-atrial fibrillation and flutter comorbidity patterns could enable proactive, preventive, and personalized healthcare.

**Methods:**

This population-based nested case-control study analyzed data from the Korean National Health Insurance Corporation (2002–2019). Adults aged ≥19 years with at least three years of recorded claims were included. Cases were individuals newly diagnosed with atrial fibrillation and flutter between 2007 and 2019 following a washout period (2002–2006). Controls were matched 1:4 using stratified random sampling. Using 5-year disease histories, BEHRT, a transformer-based model, predicted atrial fibrillation and flutter, while BERTopic identified sex-specific multimorbidity patterns. Predictive performance was evaluated using the area under the receiver operating characteristic curve (AUC).

**Results:**

BEHRT achieved an AUC of 0.80 for predicting atrial fibrillation and flutter among 600,030 participants (8,661 cases and 591,369 controls). BERTopic analysis revealed sex-specific multimorbidity patterns: aortic aneurysm, hypertensive heart disease, and chronic obstructive pulmonary disease were common in males, while Alzheimer's disease, Parkinson's disease, and rheumatic heart disease were prominent in females.

**Discussion:**

The combination of BEHRT and BERTopic demonstrated the ability to predict atrial fibrillation and flutter based on multimorbid histories while identifying distinct sex-specific disease patterns. These findings underscore the potential for artificial intelligence to enhance personalized healthcare and optimize prevention and management strategies for chronic conditions.

## 1 Introduction

Multimorbidity is becoming increasingly prevalent globally due to aging populations, lifestyle changes, and environmental factors, presenting significant challenges to healthcare systems. Given the complexity of managing multiple chronic conditions (1), a detailed understanding of multimorbidity is crucial for developing integrated and patient-centered care models (2) that improve health outcomes and reduce the burden on healthcare systems (3). Additionally, multimorbidity in an individual is not merely coincidental; one condition may contribute to the development of another. Hence, identifying non-random disease associations is crucial for developing more effective preventive and therapeutic interventions (4).

Atrial fibrillation and flutter (AFF), the most prevalent clinical arrhythmias worldwide, affecting millions of people, and the number of cases is expected to increase, particularly among older adults (5). The increasing prevalence of AFF poses challenges to healthcare systems owing to complications such as ischemic stroke, dementia, and cognitive dysfunction. These health and socioeconomic effects necessitate effective management strategies (6–8). AFF is a complex condition frequently accompanied by other health issues, highlighting the necessity for optimized care processes for patients with AFF and multimorbidity (9). Understanding the longitudinal comorbidity patterns preceding AFF could enable proactive prevention and personalized care for related conditions (10).

Artificial intelligence (AI) in disease prediction, as explored by Dahiwade et al. (11), can help address multimorbidity challenges. Machine learning (ML) techniques, such as K-nearest neighbor (KNN) and convolutional neural networks (CNN), can be used for disease prediction based on patient symptoms. These techniques enable large dataset classification and accurate disease prediction, aiding in effective multimorbidity management (12–14). Specifically, advanced deep learning techniques such as bidirectional encoder representation from transformers for electronic health records (BEHRT) (15) and BERTopic (16) can be powerful tools for identifying associations between diseases and elucidating multimorbidity development.

Using a nationally representative, large-scale claims database and applying advanced deep-learning techniques, this study explored multimorbidity patterns that precede AFF onset. BEHRT was employed to predict the incidence of AFF and multimorbidity patterns were identified using BERTopic. Transformer-based architectures, which were originally developed for Natural Language Processing tasks, were specifically adapted to capture sequential dependencies in longitudinal healthcare data. By integrating these state-of-the-art techniques with comprehensive claims data, we aimed to understand disease progression and multimorbidity dynamics and thereby help facilitate proactive preventive care and personalized management of AFF. Furthermore, AFF exhibits differences in prevalence and age of onset between the sexes (17–19). Based on these established sex-based differences, we conducted a sex-stratified analysis.

## 2 Methods

### 2.1 Data and study population

We used data from the National Health Insurance System (NHIS) sample cohort in Korea, including anonymized health information from approximately one million individuals (representing approximately 2% of the Korean population). Since the NHIS provides mandatory coverage to nearly all Korean citizens, a sample group was constructed using stratified random sampling to ensure national representativeness. Stratification was based on socioeconomic factors such as age, sex, and income level, with proportional allocation across the strata. Specifically, we used longitudinal data from adults aged ≥19 years as of 2002, tracking them from 2002 to 2019.

Individuals who died between 2002 and 2006 were excluded to ensure a minimum 5-year follow-up for all subjects. Individuals were classified as having AFF if they had an AFF diagnosed between 2007 and 2019. A 5-year washout period (2002–2006) was used to define the incidence of AFF, ensuring that the first diagnosis of AFF was truly incident. AFF cases were identified using the International Classification of Diseases, 10th Revision (ICD-10) code I48. An individual was defined as an AFF case if they had at least one hospitalization record within 1 year or at least three outpatient visit records within the same period. Controls were selected from individuals without an AFF diagnosis from the beginning of the dataset (2002) until the end of the observation period. The study population was stratified based on the starting year of the pre-disease sequence, with the controls matched to AFF patients at a 4:1 ratio. Matching was based on the starting year of the 5-year pre-disease sequence preceding the incidence of AFF. To ensure sufficient pre-diagnostic medical history and reliability of the findings, only individuals with medical utilization records for a minimum of 3 of the 5 years prior to diagnosis were included. A schematic timeline of the disease assessment process is shown in Supplementary Figure S1.

This study utilized anonymized secondary data, and thus, the participant informed consent requirement was waived by the Institutional Review Board of the Chung-Ang University (1041078-202112-HR-336-01).

### 2.2 Cohort construction overview

**Figure 1:**
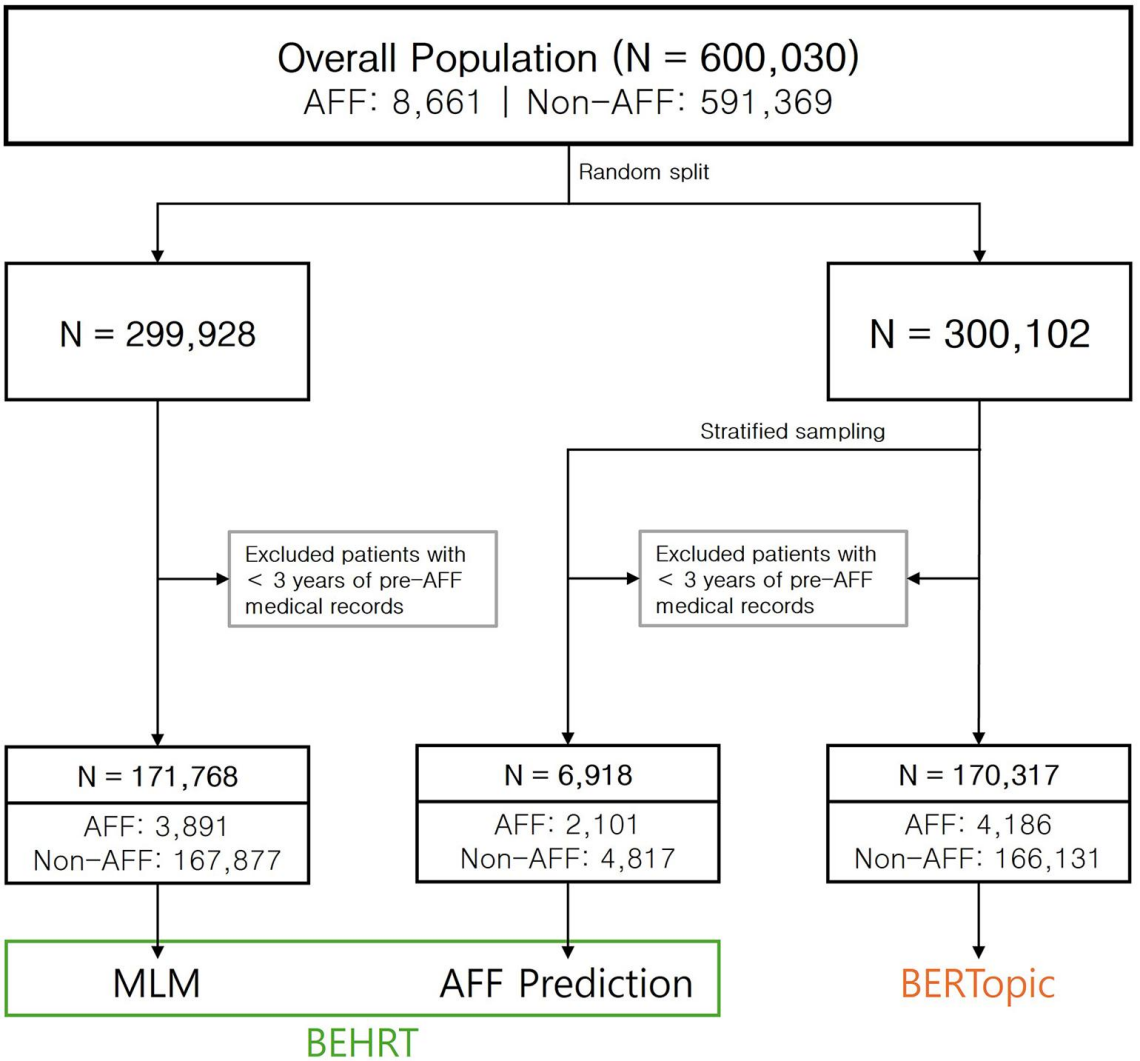
Flowchart depicting data selection and modeling process. Initial data comprised 600,030 patients divided into AFF (*n* = 8,661) and non-AFF (*n* = 591,369). BEHRT and BERTopic models were used for analysis.

To distinguish between the analytical steps and specific subsets used for each model, four specific cohorts were constructed. The overall demographic characteristics are presented in Results (Table 1). As shown in Figure 1, the total population (*n* = 600,030) was randomly divided into two equal datasets (50% each) to ensure independent development and analysis of subsets (Supplementary Table S1).

MLM Pre-training cohort: The first half (*n* = 299,928) was designated for the unsupervised Masked Language Model (MLM) task. After excluding patients with insufficient medical history (<3 years), the final MLM cohort comprised 171,768 individuals (Supplementary Table S2).
Downstream Analysis cohorts: The second half (*n* = 300,102) served as the source for both the AFF Prediction and BERTopic analyses.
  Prediction cohort: Stratified sampling was initially performed to match the cases and controls in a 1:4 ratio. However, subsequent data quality filters (requiring ≥ 3 years of continuous disease history) disproportionately excluded controls with fragmented records. This resulted in a final prediction cohort of 6,918 patients, with an observed ratio of approximately 1:2.3 (Supplementary Table S3).
  BERTopic cohort: To maximize pattern discovery, the full eligible subset was used without downsampling, resulting in 170,317 patients after exclusion (Supplementary Table S4).

**Table 1:** Overall characteristics of study population.

Variable	Cases	Controls	*p*-value
**Total**	**8,661**	**591,369**	
Sex			**<0**.**001**
Male	4,861 (56.1)	280,646 (47.5)	
Female	3,800 (43.9)	310,723 (52.5)	
Age group^a^ (years)			**<0**.**001**
19–39	864 (10.0)	267,355 (45.2)	
40–64	5,321 (61.4)	264,726 (44.8)	
over 65	2,476 (28.6)	59,288 (10.0)	

Bold values indicate statistical significance (*p* < 0.05).

Values are presented as *N* (%).

a Based on year 2002.

### 2.3 Disease history

Initially, 126 diseases, including AFF, were selected. Disease identification was based on previously published research claim definitions (20). A disease was considered to have occurred if the patient had at least one hospitalization within 1 year or if the number of outpatient visits for a specific disease reached the specified threshold during the same period (Supplementary Table S5). Following the application of these criteria, 28 diseases failed to satisfy the established standards, leading to the inclusion of 98 diseases in the final study. To analyze longitudinal multimorbidity patterns, we examined the 5-year disease history before the incidence of AFF.

### 2.4 Rationale for model integration

BEHRT and BERTopic have complementary roles. BEHRT captures temporal dependencies in a patient's disease history to predict AFF risk, whereas BERTopic identifies latent multimorbidity structures to interpret disease trajectories. Prediction alone cannot explain the clinical pathways, and BERTopic alone cannot assess risk. Together, these two models provide a unified framework that simultaneously forecasts AFF and reveals interpretable multimorbidity clusters underlying its development.

### 2.5 Statistical analysis

Through the integration of BEHRT for predictive modeling and BERTopic for disease evolution analysis elucidated the multimorbidity patterns associated with AFF. In this analysis, a unified model was used instead of sex-specific models to maintain data efficiency and robustly learn temporal dependencies.

### 2.6 Statistics and reproducibility

Data Splitting: To rigorously evaluate the prediction model, the final prediction cohort for BEHRT (*n* = 6,918) was split into three independent datasets: 60% for training, 20% for validation (used for hyperparameter tuning), and 20% for independent testing. Stratified splitting was performed to ensure that the proportions of AFF patients and controls remained consistent across all three subsets.
Reproducibility: To ensure full reproducibility, we have provided a detailed list of libraries and version numbers. The analysis relied on Python (v.3.8+) and key libraries including PyTorch, transformers, sentence transformers, UMAP, HDBSCAN, BERTopic, scikit-learn, pandas, and numpy. We pinned the package versions in a requirements.txt file and provided a complete 5-step workflow (from preprocessing to model training) in the public repository. Detailed code is available at https://github.com/skwgbobf/AFF-ai-project.

### 2.7 BEHRT for disease embedding and AFF prediction

**Figure 2:**
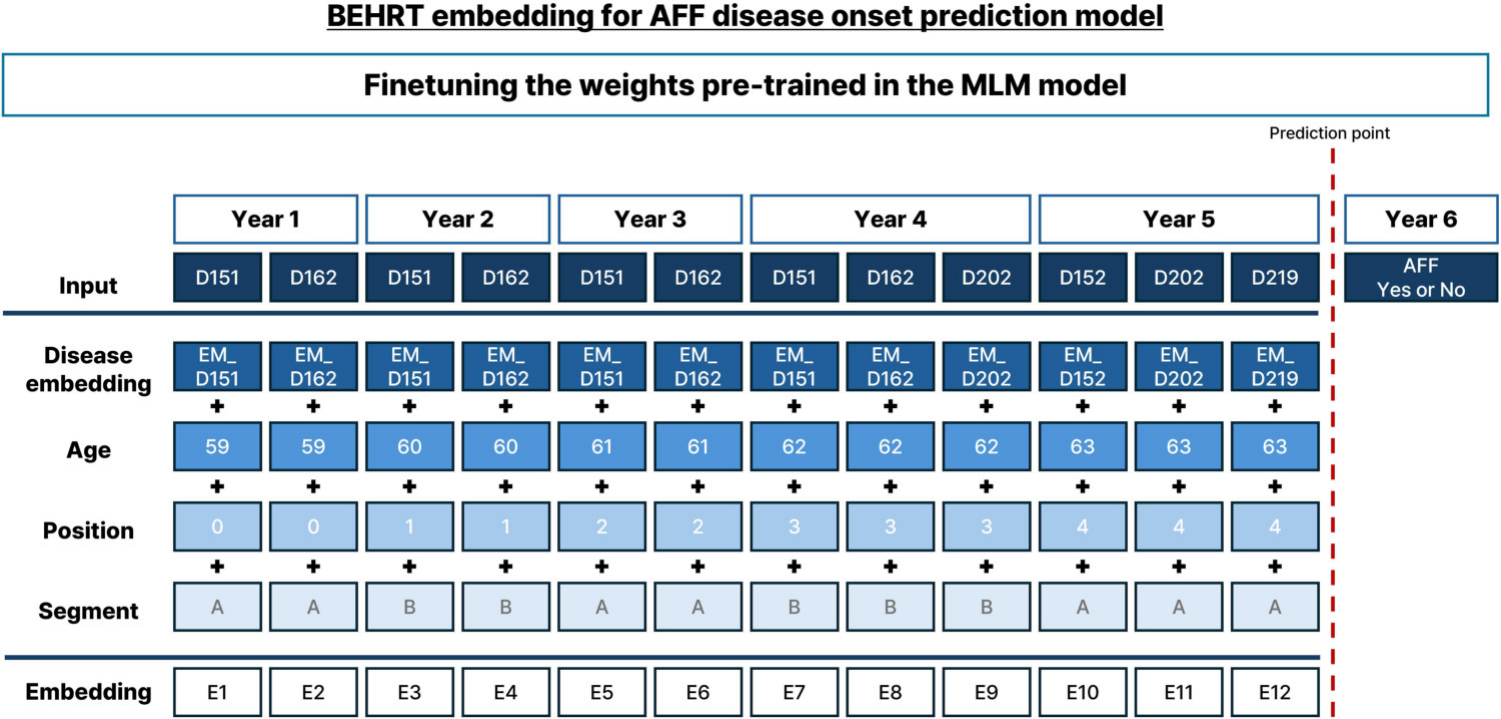
Schematic representation of embedding using BEHRT for predicting AFF onset. Fine-tuning involved pre-trained weights using a masked language model (MLM) on sequential disease codes and event markers over time. The output layer predicted AFF incidence.

The BEHRT model, which utilizes transformer-based neural networks, was designed to capture chronological and contextual information in electronic health data. As illustrated in Figure 2, the model embeddings include disease, age, segment, and position embeddings, which collectively capture the complexity of a patient's medical history.

$$\mathrm{Embedding}\;(x)=W_{e}\cdot x+Pos_{e}\cdot p(x)+Seg_{e}\cdot s(x)+Age_{e}\cdot a(x)$$

Where $\left(W_{e}\right)$ represents the disease embedding matrix, $\left(Pos_{e}\right)$ denotes the position embedding, [p(x)] indicates the positional index, $\left(Seg_{e}\right)$ corresponds to the segment embedding, [s(x)] represents the segment index, $\left(Age_{e}\right)$ refers to the age embedding, and [a(x)] indicates the age.

Disease Embeddings: Capture the specific diseases recorded in the patient's history.
Position Embedding: Encodes the temporal order of events within the patient's health record, ensuring that the model understands the sequence of disease occurrences over time.
Segment Embeddings: Differentiate between different segments of the patient's annual health records.
Age Embeddings: Encode the patient's age at each point in time.

Key Strengths of BEHRT:

Contextual Understanding: Captures the context in which diseases occur by considering the chronological order of events and the patient's age at each stage.
Pattern Recognition: Employs a MLM to identify disease progression patterns, potentially highlighting the risk factors for conditions such as AFF.
Predictive Capability: Can be fine-tuned for downstream tasks, including predicting the onset of specific diseases, by learning from pre-diagnosis patterns in historical data.
Customization: Can be tailored to specific diseases or conditions, making it a versatile research tool.

#### 2.7.1 MLM pretraining

MLM pretraining enables the model to learn from complex medical histories by predicting masked portions of the input sequence, thereby gaining a deeper understanding of the underlying patterns within the data.

We developed a foundational MLM that served as the basis for a downstream task specifically designed to predict AFF incidence. We analyzed disease patterns during the 5 years leading up to its onset.

#### 2.7.2 Predictive modeling

To predict the incidence of AFF, BEHRT analyzes the sequence of diseases diagnosed in the 5 years preceding AFF incidence. This continuous tracking of disease sequences allows the model to understand how prior conditions influence the likelihood of AFF compared with other diseases. The prediction task was formulated as a classification problem, where the model outputs the probabilities of AFF incidence by analyzing these pre-diagnosis patterns. Model performance was evaluated using the area under the receiver operating characteristic curve (AUROC). To benchmark this performance, we compared BEHRT with a Long Short-Term Memory (LSTM) model, which is a standard baseline for sequential EHR modeling.

### 2.8 Disease evolution with BERTopic

In addition to BEHRT, BERTopic has been used to map disease progression and identify patterns indicative of the onset of AFF.

#### 2.8.1 Latent health topic analysis

BERTopic was selected because it can analyze large, unstructured datasets and identify latent health topics. Unlike traditional topic modeling, which can analyze topics independently for each class, BERTopic was implemented per class to better understand how specific topics were represented across different subgroups within the dataset.

#### 2.8.2 Technical implementation

Disease sequences were represented as space-separated lists of numeric disease identifiers (e.g., “195 202 220 145”), where each integer corresponds to 1 of the 126 chronic diseases included in the vocabulary. The numeric label “138” denotes the disease index for AFF and does not indicate the vocabulary size.

These sequences were encoded using the sentence-transformer all-MiniLM-L6-v.2 model to generate fixed-length embeddings based on the disease co-occurrence patterns. For dimensionality reduction, we used UMAP (n_components = 5, min_dist = 0.0, metric = “cosine”), selected through empirical stability testing to maximize structure preservation in medical trajectories. Clustering was performed using HDBSCAN [min_cluster_size = 150, min_samples = 1 (default)] with the Euclidean metric, which is appropriate for clustering UMAP-reduced embeddings that lie on a local Euclidean manifold. Class TFIDF was used to dynamically identify the most relevant and distinct topics (or disease patterns) in this complex dataset.

## 3 Results

A total of 600,030 individuals were included in the analysis. Table 1 presents the basic characteristics of the study population, including age, sex, and age group distribution, for both AFF cases (*n* = 8,661) and controls (*n* = 591,369). The proportion of males was higher in the AFF group (56%) than in the control group (47%), whereas females were more represented among the control group (53%) than among the case group (44%) (*p* < 0.001).

When considering age groups, the majority of AFF cases were between 40 and 64 years (61%), while the largest age group among controls was 19–39 years (45%) (*p* < 0.001). Older adults (over 65 years) comprised a higher proportion of AFF cases (29%) than controls (10%).

### 3.1 Pretraining the MLM

During pretraining, a percentage of disease codes in the sequences was randomly masked, with the model trained to predict these masked codes based on the surrounding context. The model achieved an F1 score of 0.86.

### 3.2 Fine-tuning for AFF onset prediction

**Figure 3:**
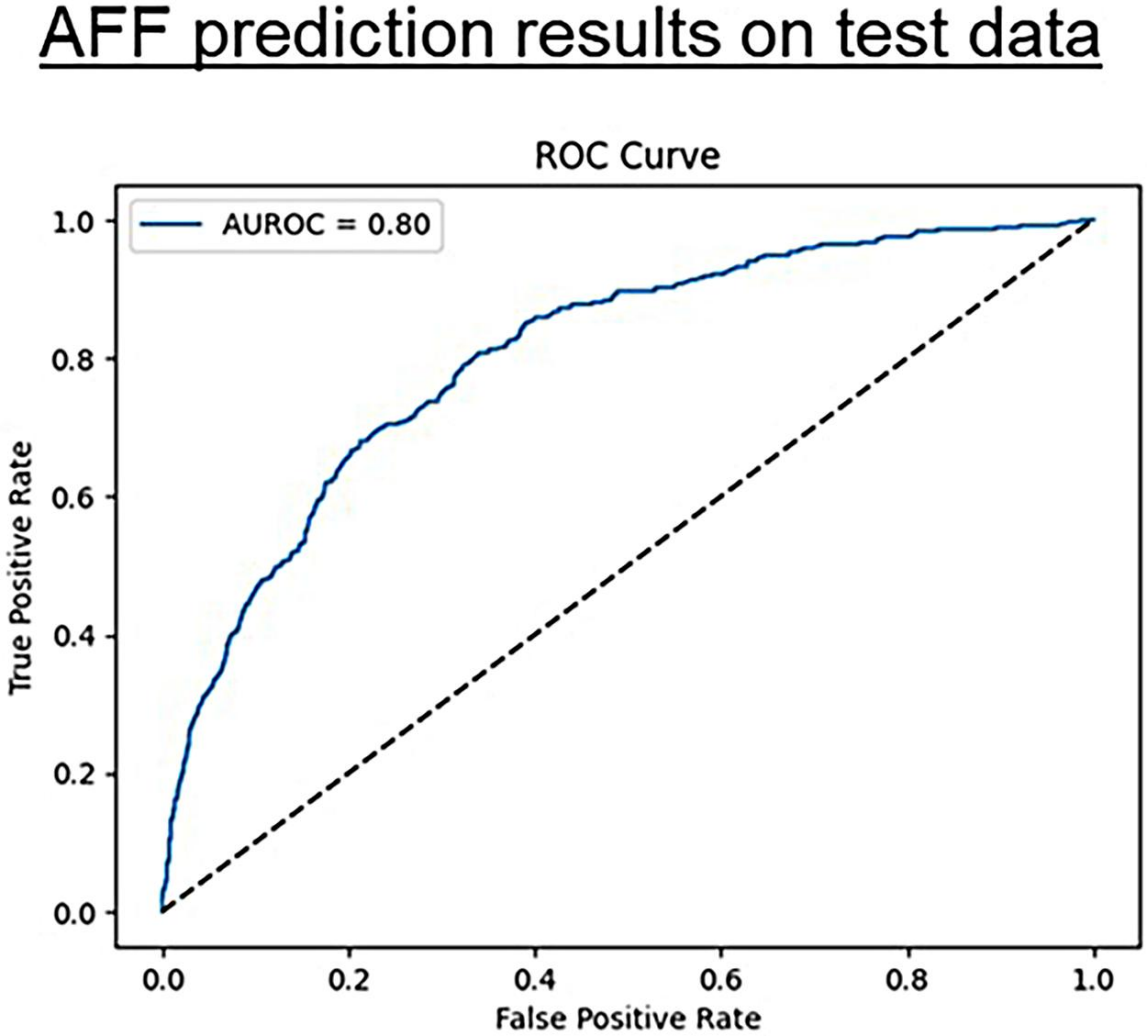
AFF prediction performance on test data. The area under the ROC curve (AUROC) is 0.80, indicating that the model has a high level of discriminatory power.

The pre-trained BEHRT model was adapted to predict the incidence of AFF using 5-year disease histories. The best AUROC achieved during fine-tuning was 0.81 (Supplementary Figure S2). The model achieved an AUROC of 0.80, with an F1 score of 0.40 and an area under the precision-recall curve of 0.57 (Figure 3), which is consistently reported as the primary performance metric. Using identical data splits, the LSTM achieved an AUROC of 0.73 (Supplementary Figure S3).

### 3.3 Disease patterns leading to AFF

**Figure 4:**
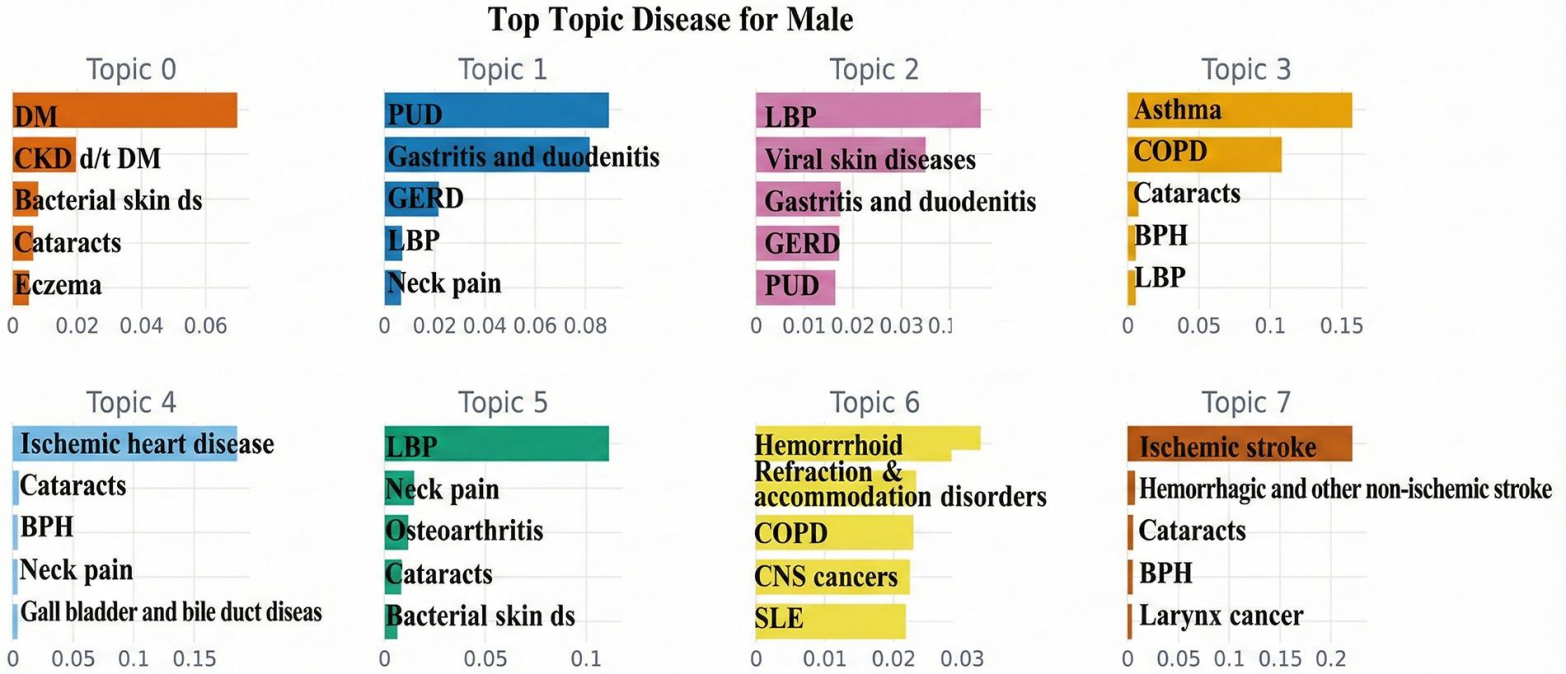
Prevalent conditions in males identified by BERTopic. The bars are sorted in descending order of their c-TF-IDF scores; terms on the left represent the most important or defining words for that topic. A higher *x*-axis value indicates a stronger association with that particular topic. Only the top 5 patterns per topic are presented. DM, diabetes mellitus; CKD d/t DM, chronic kidney disease due to DM; abscess, impetigo, and other bacterial skin diseases: bacterial skin ds; PUD, peptic ulcer disease; GERD, gastroesophageal reflux disease; LBP, low back pain; COPD, chronic obstructive pulmonary disease; BPH, benign prostatic hyperplasia; SLE, systemic lupus erythematosus; tubulointerstitial nephritis, pyelonephritisUTI, urinary tract infections; CNS, central nervous system.

**Figure 5:**
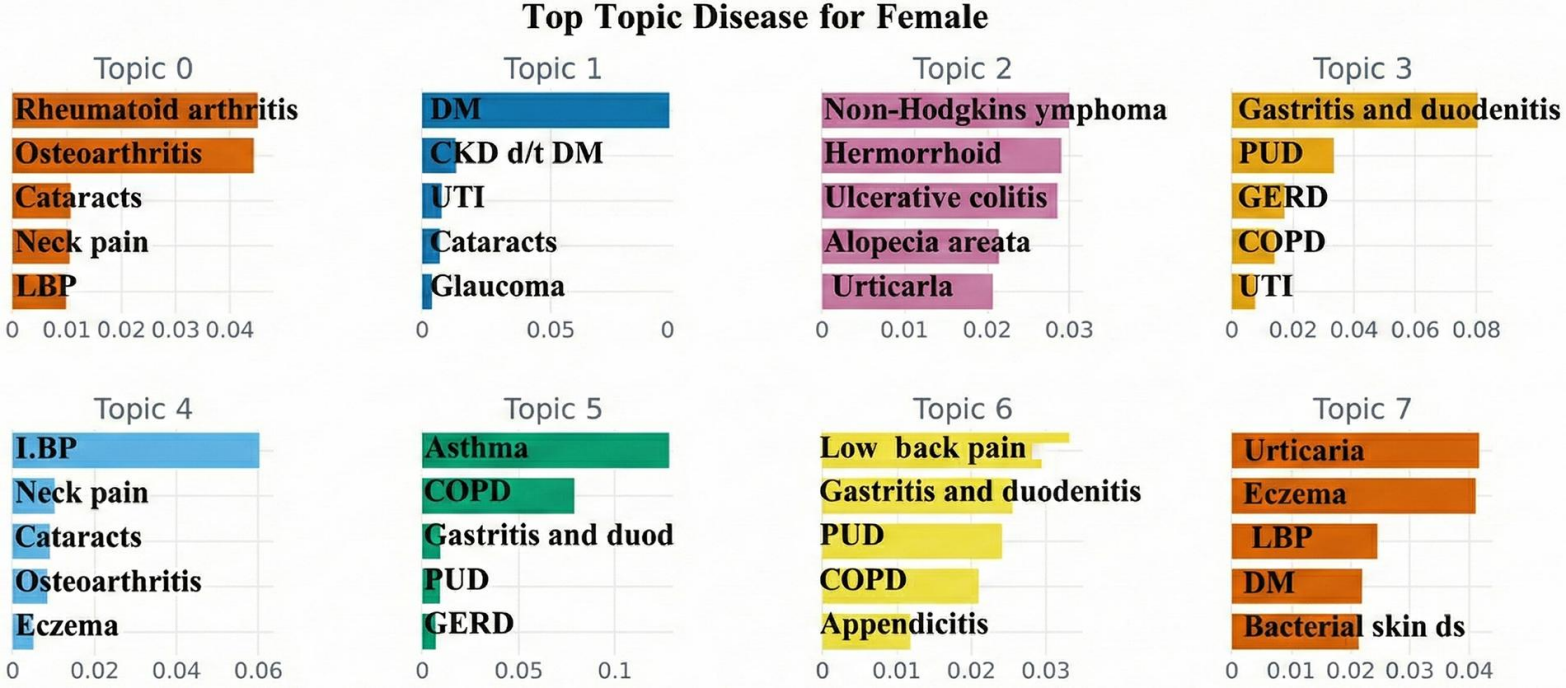
Prevalent conditions in females identified by BERTopic. The bars are sorted in descending order of their c-TF-IDF scores; terms on the left represent the most important or defining words for that topic. A higher *x*-axis value indicates a stronger association with that particular topic. Only the top 5 patterns per topic. LBP, low back pain; DM, diabetes mellitus; CKD d/t DM, chronic kidney disease due to DM; UTI, urinary tract infections; PUD, peptic ulcer disease; GERD, gastroesophageal reflux disease; COPD, chronic obstructive pulmonary disease: abscess, impetigo, and other bacterial skin diseases: bacterial skin ds.

Topic modeling identified key disease patterns for the 5 years preceding AFF compared to those without AFF. The findings for males and females are summarized in Table 2. Key conditions from BERTopic analysis are presented for both AFF and non-AFF groups, highlighting similarities and differences between sexes. Visualizations are provided in Figures 4, 5.

**Table 2:** Case–control multimorbidity patterns based on the 5-year disease history preceding AFF occurrence.

Topics	Male				Female			
	AFF (2,128 patients)		Non-AFF (76,674 patients)		AFF (2,058 patients)		Non-AFF (89,457 patients)	
	Top 5 diseases	%	Top 5 diseases	%	Top 5 diseases	%	Top 5 diseases	%
1	DM, CKD d/t DM, Bacterial skin ds, Cataracts, Eczema	10.7	DM, CKD d/t DM, Bacterial skin ds, Cataracts, Eczema	17.1	Osteoarthritis, Rheumatoid arthritis, Alzheimer's disease and other dementias, LBP, Cataracts	14.5	Rheumatoid arthritis, Osteoarthritis, Cataracts, Neck pain, LBP	12.0
2	Gastritis and duodenitis, PUD, Periodontal disease, GERD, Colon and rectum cancers	3.2	PUD, Gastritis and duodenitis, GERD, LBP, Neck pain	7.2	DM, UTI, Viral skin diseases, Decubitus ulcer, Cataracts	3.6	DM, CKD d/t DM, UTI, Cataracts, Glaucoma	6.4
3	LBP, COPD, Viral skin diseases, GERD, Gastritis and duodenitis	6.9	LBP, Viral skin diseases, Gastritis and duodenitis, GERD, PUD	5.9	Rheumatic heart disease, Parkinson's disease, Hemorrhoid, Gallbladder and biliary tract cancer, UTI	5.1	Non-Hodgkins lymphoma, hemorrhoid, Ulcerative colitis, Alopecia areata, Urticaria	5.3
4	Asthma, COPD, Cataracts, Rheumatoid arthritis, BPH	5.2	Asthma, COPD, Cataracts, BPH, LBP	4.8	Gastritis and duodenitis, PUD, COPD, GERD, Viral skin diseases	2.8	Gastritis and duodenitis, PUD, GERD, COPD, UTI	5.0
5	IHD, Aortic aneurysm, Cataracts, Hypertensive heart disease, Scabies	6.7	IHD, Cataracts, BPH, Neck pain, Gall bladder and bile duct disease	4.2	LBP, Cataracts, Osteoarthritis, Neck pain, Aortic aneurysm	4.0	LBP, Neck pain, Cataracts, Osteoarthritis, Eczema	4.9
6	LBP, Cataracts, Neck pain, Bacterial skin ds, Osteoarthritis	3.6	LBP, Neck pain, Osteoarthritis, Cataracts, Bacterial skin ds	3.9	Asthma, COPD, Endocarditis, Aortic aneurysm, PUD	3.2	Asthma, COPD, Gastritis and duodenitis, PUD, GERD	4.0
7	Rheumatic heart disease, Cardiomyopathy and myocarditis, COPD, BPH, IHD	6.2	Hemorrhoid, Refraction and accommodation disorders, CNS cancer, COPD, SLE	3.7	Alzheimer's disease and other dementias, LBP, COPD, Gastritis and duodenitis, PUD	2.4	LBP, Gastritis and duodenitis, PUD, COPD, Appendicitis	3.7
**Total**	**42.5%**		**46** **.** **8%**		**35**.**6%**		**41.3%**	

DM, Diabetes mellitus; CKD d/t DM, chronic kidney disease due to DM; abscess, impetigo, and other bacterial skin diseases; bacterial skin ds; PUD, peptic ulcer disease; GERD, gastroesophageal reflux disease; LBP, low back pain; COPD, chronic obstructive pulmonary disease; BPH, benign prostatic hyperplasia; IHD, ischemic heart disease; SLE, systemic lupus erythematosus; tubulointerstitial nephritis, pyelonephritis; UTI, urinary tract infections; brain and nervous system cancers, (central nervous system) cancer.

For males:

Topic 1: Common conditions were more prevalent in non-AFF patients.
Topic 2: Gastrointestinal diseases were observed in both groups, with periodontal disease and colon/rectal cancers primarily in AFF.
Topic 3: COPD was notably more common in AFF patients.
Topic 4: Similar combinations of diseases were seen in both AFF and non-AFF groups.
Topic 5: Aortic aneurysms and hypertensive heart disease were specific to AFF patients.
Topic 6: Both groups showed similar combinations of musculoskeletal and respiratory conditions.
Topic 7: Heart diseases, such as rheumatic heart disease and cardiomyopathy, were more common in AFF.

For females:

Topic 1: Musculoskeletal diseases were associated with Alzheimer's in AFF patients, while cataracts were common in non-AFF patients.
Topic 2: Viral skin diseases and decubitus ulcers were linked in AFF patients, whereas diabetes and eye diseases were more common in non-AFF patients.
Topic 3: Hemorrhoids and gallbladder cancer were associated with AFF patients.
Topic 4: Both groups showed similar combinations of gastrointestinal and respiratory diseases.
Topic 5: Aortic aneurysms were specific to AFF patients.
Topic 6: Respiratory diseases were combined with endocarditis and aortic aneurysms in AFF patients.
Topic 7: Alzheimer's disease was linked with gastrointestinal and musculoskeletal conditions in AFF patients.

## 4 Supplementary analysis

This subgroup analysis revealed significant differences in the proportions of diseases between the AFF and non-AFF groups for both males and females, as indicated by the Z-proportion test *p*-values (*p* < 0.0005) adjusted for multiple comparisons using the Bonferroni correction (Supplementary Tables S6, S7).

## 5 Discussion

Using AI and health informatics, particularly through BEHRT model and BERTopic analysis integration, provided novel insights into multimorbidity associated with AFF. The BEHRT model exhibited moderate predictive performance in identifying individuals at risk of AFF utilizing a 5-year longitudinal disease history. BERTopic further revealed structured and clinically significant multimorbidity patterns within these histories, identifying clusters of conditions that may contribute to the development of AFF. These multimorbidity patterns exhibited clear sex-specific differences, with distinct disease profiles emerging for males and females.

Despite the significant advancements in ML and AI, previous studies have struggled to address the unique challenges associated with multimorbidity in patients with multiple chronic conditions. A major research gap lies in the inability of the existing ML and AI approaches to effectively capture temporal dynamics and the intricate interplay between multiple chronic conditions. Traditional models, such as KNN and CNN, often struggle with temporal dependencies, high-dimensional data, and contextual understanding, which are essential for accurate disease prediction and prevention. Current methodologies, including network analysis and targeted maximum likelihood estimation (TMLE) (7), have certain limitations. Network analysis, while effective in revealing disease associations, often neglects the sequence and timing of disease occurrence, which are crucial for early detection and intervention. Although powerful for estimating causal effects, TMLE may not be optimal for uncovering complex multimorbidity patterns in high-dimensional datasets owing to the need to pre-specify confounders and causal pathways.

To address this gap, we have developed advanced models to understand the sequential progression of various diseases. Models, such as BEHRT, which can process sequential data and capture contextual information, are promising solutions. This model demonstrated the superior capability of the transformer architecture to capture complex longitudinal dependencies compared with the traditional recurrent network. BERTopic presents a unique opportunity for multimorbidity pattern identification by analyzing disease narratives and capturing the temporal evolution of multimorbidity patterns. The interpretability of the BERTopic output further enhances its clinical utility. This study demonstrated the potential of advanced AI models for identifying disease patterns and predicting AFF incidence, although further research is required to refine these models.

Our results together with the existing knowledge of AFF and its comorbidities confirmed numerous well-established clinical associations (21–23) and highlighted new potential connections. While the relationship between AFF and cardiovascular disease had already been shown (24), our study revealed patterns such as the significant co-occurrence of aortic aneurysms preceding AFF. Previous studies have demonstrated the co-occurrence of thoracic aortic aneurysms with AFF, highlighting an elevated cardiovascular risk and a higher likelihood of thromboembolic events (25).

Additionally, this study highlighted the known associations between AFF and diabetes as well as between AFF and CKD, with both recognized as risk factors for cardiovascular complications (26–28).

Our findings support earlier research that suggest COPD insignificantly contributes to cardiac remodeling and increased atrial strain, predisposing individuals to atrial arrhythmia (29). Expert reviews further emphasize the significance of recognizing COPD as a major contributor to AFF, with mechanisms such as hypoxia, hypercapnia, and cardiac remodeling central to atrial arrhythmia development (30). Thus, COPD must be considered a critical factor in AFF management.

Similarly, the association between atopic diseases, particularly asthma, and AFF underscores the shared inflammatory pathways, highlighting the significance of a multidisciplinary approach to managing AFF (31).

Moreover, neurodegenerative diseases such as Alzheimer's were more prevalent in females, suggesting a potential role for cognitive decline in the development of AFF. This relationship may be bidirectional, where AFF increases the risk of stroke and cognitive decline, and preexisting neurological deficits may also predispose individuals to AFF (32–34).

These results suggest a broader spectrum of comorbidities in patients with AFF, offering new insights into disease interactions that may influence AFF progression and management. While further research is required to validate these findings, this enhanced understanding has important implications for improving multimorbidity prevention, enabling more personalized intervention strategies for patients with AFF, and optimizing patient care, healthcare systems, and health policies.

Comprehensive studies that focus on the epidemiology of multimorbidity and its impact on healthcare processes should be conducted. Establishing clear conceptual frameworks for understanding multimorbidity alongside the exploration of collaborative, patient-centered care models tailored to the complex needs of patients is crucial.

This study had several notable strengths. Deep learning was used to explore the complex clinical patterns associated with AFF—a relatively rare approach in multimorbidity research. BEHRT and BERTopic are powerful tools for identifying disease patterns that conventional methods fail to reveal. Moreover, the analysis leveraged nationwide population-level data from Korea to ensure the findings were robust and representative. Furthermore, by encompassing all major chronic diseases, this study offers a comprehensive view of the multimorbidity landscape, expanding on previous research that focused on a narrower range of conditions. This integrative approach offers a more nuanced understanding of the clinical patterns and disease progression in patients with AFF, particularly highlighting sex differences in disease patterns and risks. Additionally, a specific consideration in this study was the use of a disease vocabulary consisting of 126 chronic disease groups. Although this vocabulary is smaller than that of some raw national datasets, this curated dimensionality reduces code sparsity and rare-code noise. Consequently, the BEHRT model maintained a robust predictive performance (AUROC = 0.80) by focusing on stable disease signals, whereas BERTopic generated denser, more clinically interpretable multimorbidity clusters by avoiding fragmentation, which is often caused by raw ICD-10 codes.

Despite these strengths, this study had several limitations. Reliance on insurance claims data may introduce bias, as diseases are coded based on diagnostic information, which may not always accurately reflect a patient's true health status. This approach can lead to misclassification or underreporting of certain conditions. Additionally, although this study identified a strong association between AFF and various comorbidities, causality was not established. The observed relationships between AFF and comorbidities may represent correlations rather than direct causal relationships. Despite the BEHRT model's overall discriminative performance in this study, its relatively low F1 score and moderate area under the precision-recall curve suggest that the model may require further tuning to improve precision and recall, particularly in reducing false negatives and increasing true positives. Finally, data limitations precluded distinction between valvular and non-valvular AFF subtypes. Future research should integrate additional clinical data and employ more advanced methodologies to better explore the causal relationships.

For future improvements, integrating medication usage and lifestyle factors to evaluate patient health holistically would be beneficial. Extending predictive tasks to areas such as hospital readmission can further enhance the clinical utility of these models. Furthermore, as this study was based on data from a Korean population, future research should include more diverse populations to develop a global understanding of AFF and its associated multimorbidity patterns.

In conclusion, to our knowledge, this is the first study to apply these advanced methodologies to identify comorbidity patterns associated with AFF by comprehensively analyzing previous diseases associated with AFF. In males, cardiovascular disease and COPD were identified as possible contributors to AFF development, whereas in females, neurodegenerative conditions including Alzheimer's disease and other cardiovascular diseases played a similar role. By integrating AI tools, such as the BEHRT model and BERTopic analysis, we revealed novel multimorbidity patterns and disease pathways, offering valuable insights for more personalized intervention strategies.

## Data Availability

The original contributions presented in the study are included in the article/Supplementary Material, further inquiries can be directed to the corresponding author/s.
