# Supplementary material for: Predicting atrial fibrillation and flutter using BEHRT and identifying multimorbidity patterns using BERTopic
Source: Front Digit Health. 2026 Feb 5;8:1722338. doi: 10.3389/fdgth.2026.1722338 (PMC12917773; doi:10.3389/fdgth.2026.1722338)
Supplement: Supplementary file 1 [file Datasheet1.docx]

Supplementary Material

- 1. **Supplementary Tables**

**Supplementary Table 1.** Overall Study Population (Population Table)

| **Variable** | **Value** |
| --- | --- |
| Total population | 600,030 |
| AFF | 8,661 (1.44%) |
| Non-AFF | 591,369 (98.56%) |
| Inclusion criteria | ≥5-year disease history in EHR |
| Exclusions | None at this stage |
| Purpose | Base population for all downstream cohorts |

**Supplementary Table 2.** BEHRT Pre-Training (MLM) Cohort

| **Variable** | **Value** |
| --- | --- |
| Sampling method | Random 50% split from full population |
| Initial 50% Sample cohort size | 299,928 |
| Final Cohort for MLM | 171,768 |
| AFF (incident) | 3,891 |
| Non-AFF | 167,877 |
| Exclusions | Patients with < 3 years of pre-AFF medical records |

**Supplementary Table 3.** BEHRT AFF Prediction Cohort

| **Variable** | **Value** |
| --- | --- |
| Initial sampled cohort | 300,102 |
| Final AFF prediction cohort  (After stratified sampling) | 6,918 |
| AFF (incident) | 2,101 |
| Non-AFF | 4,817 |
| Sampling method | Stratified sampling |
| Exclusion criteria | Prevalent AFF; insufficient history; incomplete records |
| Prediction input window | 5-year pre-index disease history |

**Supplementary Table 4.** BERTopic Cohort Summary

| **Group** | **Total n** | **Non-AFF (n)** | **AFF (n)** |
| --- | --- | --- | --- |
| Total | 170,317 | 166,131 | 4,186 |
| Male | 78,802 | 76,674 | 2,128 |
| Female | 91,515 | 89,457 | 2,058 |

To ensure a 5-year washout period, we restricted the initial pool to patients whose medical history records commenced prior to 2015, with a follow-up duration of at least 3 years.

**Supplementary Table 5.** Claims-based disease definitions and prevalence in the overall study population(n = 600,030)

| **Disease** | **Inpatient**  **(n)** | **Outpatient (n)** | **Incidence rate (%)** |
| --- | --- | --- | --- |
| Low back pain | 1 | 5 | 38.80 |
| Gastritis and duodenitis | 1 | 3 | 23.36 |
| Osteoarthritis | 1 | 5 | 22.88 |
| Gastroesophageal reflux disease | 1 | 3 | 19.40 |
| Chronic obstructive pulmonary disease | 1 | 3 | 17.15 |
| Peptic ulcer disease | 1 | 3 | 17.12 |
| Cataracts | 1 | 5 | 15.23 |
| Diabetes mellitus | 1 | 3 | 15.05 |
| Eczema | 1 | 5 | 14.17 |
| Neck pain | 1 | 3 | 13.06 |
| Abscess, impetigo, and other bacterial skin diseases | 1 | 3 | 10.51 |
| Urticaria | 1 | 3 | 10.41 |
| Tubulointerstitial nephritis, pyelonephritis, and urinary tract infections | 1 | 5 | 9.65 |
| Fungal skin diseases | 1 | 5 | 9.50 |
| Asthma | 1 | 3 | 9.02 |
| Hemorrhoid | 1 | 5 | 8.77 |
| Benign prostatic hyperplasia | 1 | 3 | 8.54 |
| Ischemic stroke | 1 | 3 | 8.01 |
| Ischemic heart disease | 1 | 3 | 7.48 |
| Cirrhosis of the liver | 1 | 3 | 6.98 |
| Gall bladder and bile duct disease | 1 | 2 | 4.86 |
| Hypertensive heart disease | 1 | 3 | 4.81 |
| Varicose veins of lower extremities | 1 | 1 | 4.69 |
| Appendicitis | 1 | 1 | 4.39 |
| Glaucoma | 1 | 3 | 4.12 |
| Urolithiasis | 1 | 3 | 3.93 |
| Cellulitis | 1 | 3 | 3.79 |
| Tension-type headache | 1 | 3 | 3.68 |
| Rheumatoid arthritis | 1 | 3 | 2.98 |
| Viral skin diseases | 1 | 3 | 2.92 |
| Refraction and accommodation disorders | 1 | 3 | 2.77 |
| Migraine | 1 | 3 | 2.59 |
| Gout | 1 | 3 | 2.38 |
| Urinary incontinence | 1 | 2 | 2.07 |
| Pruritus | 1 | 3 | 2.01 |
| Stomach cancer | 1 | 2 | 1.89 |
| Colon and rectum cancers | 1 | 2 | 1.83 |
| Peripheral vascular disease | 1 | 3 | 1.81 |
| Thyroid cancer | 1 | 2 | 1.77 |
| Alzheimer's disease and other dementias | 1 | 2 | 1.76 |
| Hemorrhagic and other non-ischemic stroke | 1 | 3 | 1.64 |
| Alopecia areata | 1 | 3 | 1.64 |
| Atrial fibrillation and flutter | 1 | 3 | 1.44 |
| Trachea, bronchus and lung cancers | 1 | 2 | 1.33 |
| Pancreatitis | 1 | 2 | 1.31 |
| Paralytic ileus and intestinal obstruction without hernia | 1 | 2 | 1.25 |
| Psoriasis | 1 | 3 | 1.24 |
| Inguinal or femoral hernia | 1 | 2 | 1.19 |
| Liver cancer | 1 | 2 | 1.04 |
| Parkinsons disease | 1 | 3 | 0.98 |
| Chronic kidney disease due to hypertension | 1 | 3 | 0.92 |
| Scabies | 1 | 2 | 0.64 |
| Chronic kidney disease unspecified | 1 | 3 | 0.62 |
| Decubitus ulcer | 1 | 2 | 0.56 |
| Chronic kidney disease due to diabetes mellitus | 1 | 3 | 0.54 |
| Macular degeneration | 1 | 3 | 0.49 |
| Interstitial lung disease and pulmonary sarcoidosis | 1 | 2 | 0.39 |
| Bladder cancer | 1 | 2 | 0.36 |
| Gallbladder and biliary tract cancer | 1 | 2 | 0.35 |
| Cardiomyopathy and myocarditis | 1 | 3 | 0.35 |
| Periodontal disease | 1 | 5 | 0.35 |
| Mouth cancer | 1 | 2 | 0.33 |
| Pancreatic cancer | 1 | 2 | 0.33 |
| Acne vulgaris | 1 | 3 | 0.32 |
| Brain and nervous system cancers | 1 | 2 | 0.29 |
| Aortic aneurysm | 1 | 2 | 0.28 |
| Kidney cancer | 1 | 2 | 0.27 |
| Non-melanoma skin cancer | 1 | 2 | 0.26 |
| Rheumatic heart disease | 1 | 3 | 0.26 |
| Dental caries | 1 | 3 | 0.25 |
| Non-Hodgkins lymphoma | 1 | 2 | 0.24 |
| Ulcerative colitis | 1 | 3 | 0.24 |
| Benign neoplasm of brain and other parts of central nervous system | 1 | 2 | 0.22 |
| Vascular disorders of intestine | 1 | 2 | 0.22 |
| Acute glomerulonephritis | 1 | 3 | 0.18 |
| Esophageal cancer | 1 | 2 | 0.15 |
| Leukemia | 1 | 2 | 0.15 |
| Bone and connective tissue cancer | 1 | 2 | 0.13 |
| Multiple myeloma | 1 | 2 | 0.11 |
| Systemic lupus erythematosus(SLE) | 1 | 3 | 0.11 |
| Larynx cancer | 1 | 2 | 0.10 |
| Crohns disease | 1 | 3 | 0.10 |
| Cancer of other part of pharynx and oropharynx | 1 | 2 | 0.08 |
| Malignant melanoma of skin | 1 | 2 | 0.07 |
| Pneumoconiosis | 1 | 2 | 0.06 |
| Breast cancer | 1 | 2 | 0.04 |
| Nasopharynx cancer | 1 | 2 | 0.04 |
| Ovarian cancer | 1 | 2 | 0.03 |
| Multiple sclerosis | 1 | 2 | 0.02 |
| Uterine cancer | 1 | 2 | 0.01 |
| Prostate cancer | 1 | 2 | 0.01 |
| Hodgkins disease | 1 | 2 | 0.01 |
| Endocarditis | 1 | 3 | 0.01 |
| Testicular cancer | 1 | 2 | 0.00 |
| Schizophrenia | 1 | 1 | 0.00 |
| Thalassemias | 1 | 3 | 0.00 |
| Sickle cell disorders | 1 | 2 | 0.00 |
| G6PD deficiency | 1 | 3 | 0.00 |
| Alcohol use disorders | 1 | 2 | - |
| Amphetamine use disorders | 1 | 1 | - |
| Anorexia nervosa | 1 | 2 | - |
| Aspergers syndrome | 1 | 2 | - |
| Attention-deficit hyperactivity disorder | 1 | 3 | - |
| Autism | 1 | 2 | - |
| Bipolar affective disorder | 1 | 1 | - |
| Borderline personality disorder | 1 | 3 | - |
| Bulimia nervosa | 1 | 2 | - |
| Cannabis use disorders | 1 | 1 | - |
| Cervical cancer | 1 | 2 | - |
| Cocaine use disorders | 1 | 1 | - |
| Conduct disorder | 1 | 1 | - |
| Dysthymia | 1 | 1 | - |
| Endometriosis | 1 | 2 | - |
| Epilepsy | 1 | 3 | - |
| Female infertility | 1 | 3 | - |
| Genital prolapse | 1 | 2 | - |
| Idiopathic intellectual disability | 1 | 2 | - |
| Major depressive disorders | 1 | 3 | - |
| Male infertility | 1 | 3 | - |
| Obsessive-compulsive disorder | 1 | 2 | - |
| Opioid use disorders | 1 | 1 | - |
| Panic disorder | 1 | 2 | - |
| Polycystic ovarian syndrome | 1 | 3 | - |
| Post-traumatic stress disorder | 1 | 2 | - |
| Premenstrual syndrome | 1 | 3 | - |
| Uterine fibroids | 1 | 2 | - |

Only the incidence rates for the 98 diseases, as defined by the established disease identification criteria in the analysis, were computed and included in the final analysis.

**Supplementary Table 6.** Distribution of diseases in males: AFF group vs. non-AFF group

| **Diseases** | **Cases**  **(N=2,128)** | | **Controls**  **(N=76,674)** | | ***P*-value** |
| --- | --- | --- | --- | --- | --- |
|  | N | % | N | % |  |
| Periodontal disease | 12 | 0.07 | 282 | 0.06 | 0.647 |
| Colon and rectum cancers | 161 | 0.99 | 5,860 | 1.34 | <0.0005 |
| Chronic obstructive pulmonary disease | 832 | 5.10 | 16,327 | 3.70 | <0.0005 |
| Rheumatoid arthritis | 72 | 0.40 | 2,766 | 0.60 | 0.003 |
| Aortic aneurysm | 40 | 0.25 | 448 | 0.10 | <0.0005 |
| Hypertensive heart disease | 526 | 3.20 | 11,349 | 2.60 | <0.0005 |
| Rheumatic heart disease | 139 | 0.90 | 534 | 0.10 | <0.0005 |
| Cardiomyopathy and myocarditis | 146 | 0.90 | 771 | 0.20 | <0.0005 |
| Benign prostatic hyperplasia | 1,769 | 10.9 | 28,981 | 6.60 | <0.0005 |
| Ischemic heart disease | 1,220 | 7.50 | 21,208 | 4.80 | <0.0005 |

A Z-test for proportions with Bonferroni correction for multiple comparison was applied: the Z-Test results reject the null hypothesis when the p-value is less than 0.0005, after applying Bonferroni correction. This indicates that the proportions of these diseases significantly differ between the AFF and non-AFF groups.

**Supplementary Table 7.** Distribution of diseases in females: AFF group vs. non-AFF group

| **Diseases** | **Cases**  **(N=2,058)** | | **Controls**  **(N=89,457)** | | ***P*-value** |
| --- | --- | --- | --- | --- | --- |
|  | **N** | **%** | **N** | **%** |  |
| Alzheimer's disease and other dementias | 85 | 0.50 | 929 | 0.20 | <0.0005 |
| Viral skin diseases | 43 | 0.27 | 1,586 | 0.31 | 0.345 |
| Decubitus ulcer | 10 | 0.06 | 244 | 0.05 | 0.406 |
| Rheumatic heart disease | 299 | 1.80 | 1,129 | 0.20 | <0.0005 |
| Parkinsons disease | 116 | 0.70 | 2,477 | 0.50 | <0.0005 |
| Aortic aneurysm | 25 | 0.20 | 323 | 0.10 | <0.0005 |
| Endocarditis | 5 | 0.03 | 5 | 0.00 | <0.0005 |

A Z-test for proportions with Bonferroni correction for multiple comparison was applied: the Z-Test results reject the null hypothesis when the p-value is less than 0.0005, after applying Bonferroni correction. This indicates that the proportions of these diseases significantly differ between the AFF and non-AFF groups.

## **Supplementary Figures**


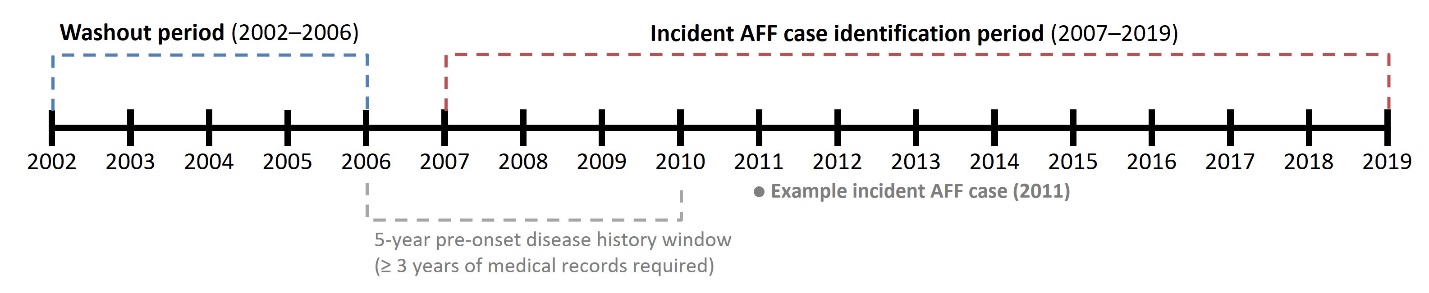


**Supplementary Figure 1.** Schematic timeline of disease assessment and cohort construction. The gray text indicates an example of an incident AFF case and the corresponding 5-year pre-onset disease history window.


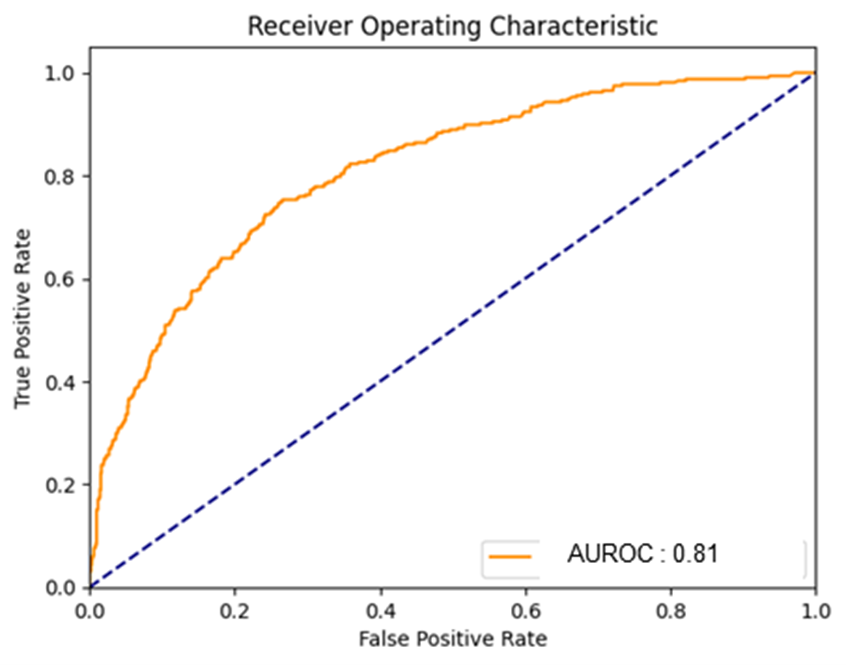


**Supplementary Figure 2.** Receiver operating characteristic (ROC) curve showing AUROC of 0.81 for predicting AFF using BEHRT. Data were split into 60% training, 20% validation, and 20% testing sets, with AFF vs. non-AFF groups balanced by participant counts. Statistical tests for sex distribution (two-proportion Z-Test, p-value = 0.42) and age means (t-Test, *p*-value = 0.86) indicate no significant differences between training and testing groups.


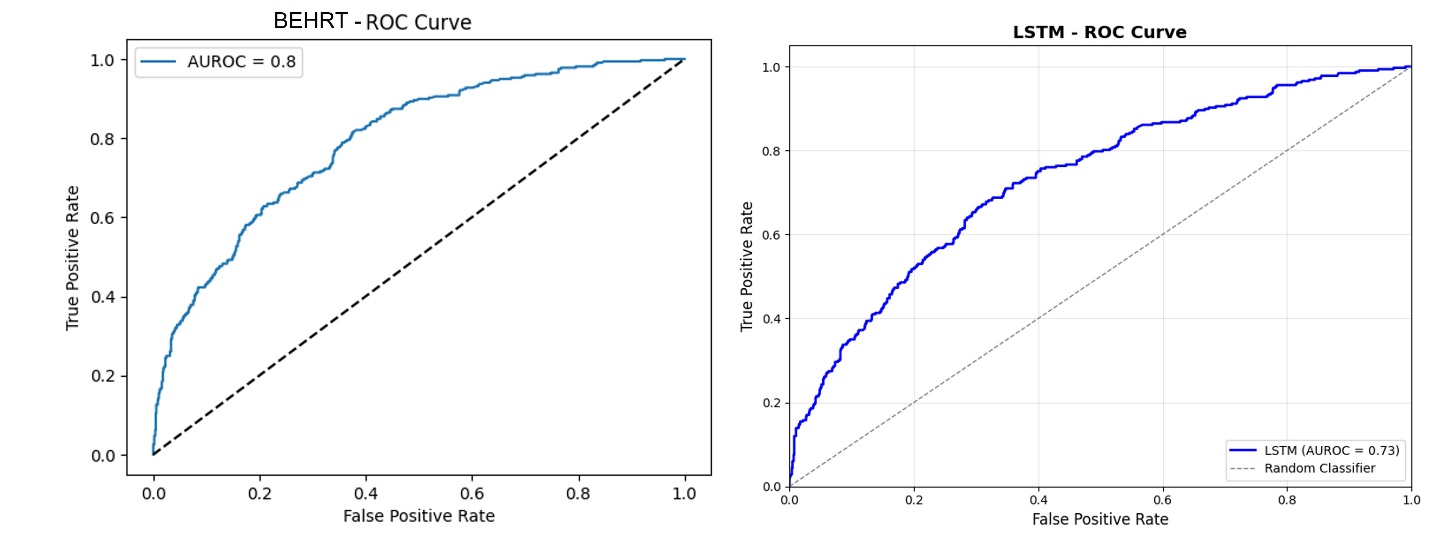


**Supplementary Figure 3.** Performance comparison between BEHRT and LSTM models for predicting AFF. The BEHRT model (left) achieved an AUROC of 0.80, whereas the baseline LSTM model (right) achieved an AUROC of 0.73. This comparison demonstrates that the transformer-based BEHRT architecture provides higher predictive performance for capturing complex disease trajectories compared to traditional recurrent models.
